# Effects and Related Mechanisms of the Senolytic Agent ABT-263 on the Survival of Irradiated A549 and Ca9-22 Cancer Cells

**DOI:** 10.3390/ijms222413233

**Published:** 2021-12-08

**Authors:** Kota Sato, Soichiro Iwasaki, Hironori Yoshino

**Affiliations:** 1 Department of Radiological Technology, Hirosaki University School of Health Sciences, Hirosaki 036-8564, Aomori, Japan; h18m2220@hirosaki-u.ac.jp (K.S.); h18m2202@hirosaki-u.ac.jp (S.I.); 2 Department of Radiation Science, Hirosaki University Graduate School of Health Sciences, Hirosaki 036-8564, Aomori, Japan

**Keywords:** senolytic agent, ABT-263, ionizing radiation, senescence-like cells, cell survival, caspase, SA-β-gal activity

## Abstract

Senolytic agents eliminate senescent cells and are expected to reduce senescent cell-mediated adverse effects in cancer therapy. However, the effects of senolytic agents on the survival of irradiated cancer cells remain unknown. Here, the effects of the senolytic agent ABT-263 on the survival of irradiated A549 and Ca9-22 cancer cells were investigated. ABT-263 was added to the culture medium after irradiation. SA-β-gal activity and cell size, which are hallmarks of cell senescence, were evaluated using a flow cytometer. The colony-forming assay and annexin V staining were performed to test cell survival. We first confirmed that radiation increased the proportion of cells with high SA-β-gal activity and that ABT-263 decreased it. Of note, ABT-263 decreased the survival of irradiated cancer cells and increased the proportion of radiation-induced annexin V^+^ cells. Furthermore, the caspase inhibitor suppressed the ABT-263-induced decrease in the survival of irradiated cells. Intriguingly, ABT-263 decreased the proportion of SA-β-gal low-activity/large cells in the irradiated A549 cells, which was recovered by the caspase inhibitor. Together, these findings suggest that populations maintaining the ability to proliferate existed among the irradiated cancer cells showing senescence-related features and that ABT-263 eliminated the population, which led to decreased survival of irradiated cancer cells.

## 1. Introduction

In normal mammalian somatic cells, the telomere DNA at the end of each chromosome shortens with each cell division and after a certain number of divisions, cell proliferation irreversibly stops (replicative senescence) [1]. This phenomenon, known as cellular senescence, was discovered by the American anatomist Leonard Hayflick in 1961 [2]. In addition to mitotic lifespan, some stressors including ionizing radiation and chemotherapy induce cellular senescence [3]. Cellular senescence is characterized by morphological changes such as enlargement and flattening, high-activity of senescence-associated β-galactosidase (SA-β-gal), and activation of signaling pathways involved with regulation of cell cycle arrest [4]. Furthermore, recent evidence has shown that senescent cells secrete various bioactive substances such as inflammatory cytokines and proteases [5], which is known as the senescence-associated secretory phenotype (SASP). These secreted substances induce inflammation of the surrounding cells and tissues [6].

From the viewpoint of cessation of cell division, cellular senescence is considered a cancer suppression mechanism. However, recent studies have found that senescent cells induced by aging or stress accumulate in tissues and organs and cause adverse effects such as tissue failure and tumorigenicity, in part, through the SASP [6,7]. Therefore, regulation of cellular senescence, elimination of senescent cells, and anti-SASP factors are thought to be effective for the prevention and treatment of senescent cell-mediated age-related diseases and adverse effects [8,9,10].

Senolytic agents are drugs that can selectively eliminate senescent cells, and are expected to treat age-related diseases and undesirable effects induced by senescent cells [10]. So far, several senolytic agents such as natural compounds (e.g., quercetin and fiestin) and targeted therapeutics (e.g., ABT-263) have been identified [10,11]. Targeted therapeutics mainly target anti-apoptotic factors because senescent cells are resistant to cell death via upregulated expression of anti-apoptotic proteins. ABT-263, also known as Navitoclax, is an inhibitor of Bcl-2/Bcl-xl and can eliminate senescent cells in irradiated normal cells thorough the induction of apoptosis [12]. In addition, oral administration of ABT-263 was reported to deplete senescent cells accumulated in mouse tissues including bone marrow after total body irradiation and ameliorate hematopoietic impairments [13].

It is thought that cancer therapy such as radiation therapy induces senescence-like cells in both normal and tumor tissues. The induction of cellular senescence in cancer cells is considered beneficial in terms of arresting the proliferation of cancer cells. In fact, induction of senescence-like cells in cancer by radiotherapy and anticancer drugs is reported to partially contribute to antitumor effects [14,15]. The elimination of senescent cells from normal tissues is expected to be beneficial for the prevention and treatment of senescent cell-mediated adverse effects in cancer therapy [16]. However, the effects of the elimination of senescence-like cancer cells on the antitumor effects of cancer therapies remain unknown.

Therefore, the aim of the present study was to investigate the effects of the senolytic agent ABT-263 on the survival of irradiated cancer cells as well as those with senescence-related features. The results showed that ABT-263 decreased the survival of irradiated cancer cells. Intriguingly, the caspase inhibitor Z-VAD recovered the ABT-263-induced decrease in the survival of irradiated cancer cells and the proportion of SA-β-gal low-activity/large cells. Together, these results suggest that among the irradiated cancer cells showing senescence-related features, ABT-263 eliminates cell populations possessing proliferative ability, resulting in decreased survival of irradiated cancer cells. In addition, it is suggested that SA-β-gal low-activity/large cells are a potential candidate of the cell population.

## 2. Results

### 2.1. Effects of ABT-263 on the Population of Cells with High SA-β-Gal Activity in Irradiated Cancer Cells

**Figure 1:**
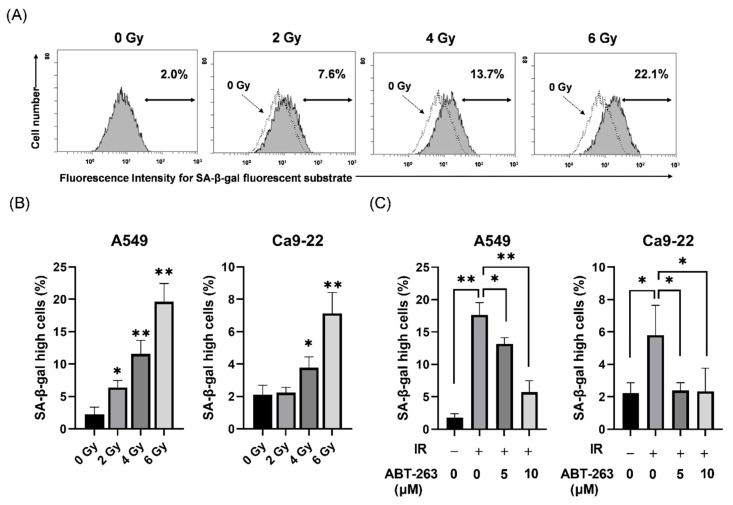
Effect of ABT-263 on populations of SA-β-gal high-activity cells in irradiated cancer cells. (**A**) X-irradiated A549 cells were cultured for 48 h and SA-β-gal activity was analyzed using the Senescence β-Galactosidase Activity Assay Kit. Representative histograms of SA-β-gal activity in A549 cells are shown. The dotted line shows the results of non-irradiated cells and the inset numbers show the percentage of SA-β-gal high-activity cells. (**B**) X-irradiated A549 and Ca9-22 cells were cultured for 48 h prior to analysis of SA-β-gal activity. The results are shown as the percentage of SA-β-gal high-activity cells. (**C**) A549 and Ca9-22 cells irradiated at 6 Gy were cultured for 24 h prior to the addition of ABT-263 to the culture. After culturing (48 h for A549 and 24 h for Ca9-22), SA-β-gal activity was analyzed. The results are shown as the percentage of cell with high SA-β-gal activity. Data are presented as the mean ± standard deviation (SD) of independent experiments. * *p* < 0.05, ** *p* < 0.01.

First, the effects of ABT-263 on cell populations with high SA-β-gal activity, which is a well-known senescence-related feature, were investigated in irradiated cancer cells. As shown in Figure 1, SA-β-gal activity was higher in irradiated A549 cells compared to non-irradiated cells (Figure 1A) and that X-ray irradiation significantly increased the proportions of both A549 and Ca9-22 cells with high SA-β-gal activity in a dose-dependent manner (Figure 1B). Since irradiation at 6 Gy stably induced cells with high SA-β-gal activity, 6 Gy was selected for most subsequent analyses.

When ABT-263 (5 or 10 μM) was added to the culture at 24 h after 6 Gy-irradiation, the proportions of A549 and Ca9-22 cells with high SA-β-gal activity were significantly decreased (Figure 1C). These results suggest that ABT-263 suppressed the proliferation of radiation-induced senescence-like cells. In addition, 10 μM ABT-263 effectively reduced the proportion of A549 cells with high SA-β-gal activity. However, ABT-263 significantly decreased the colony-forming ability of non-irradiated A549 cells, but not non-irradiated Ca9-22 cells (Figure S1). Therefore, in the following experiments, 5 and 10 μM ABT-263 were mainly used for A549 and Ca9-22 cells, respectively.

### 2.2. Effects of ABT-263 on the Cell Survival of Irradiated Cancer Cells

**Figure 2:**
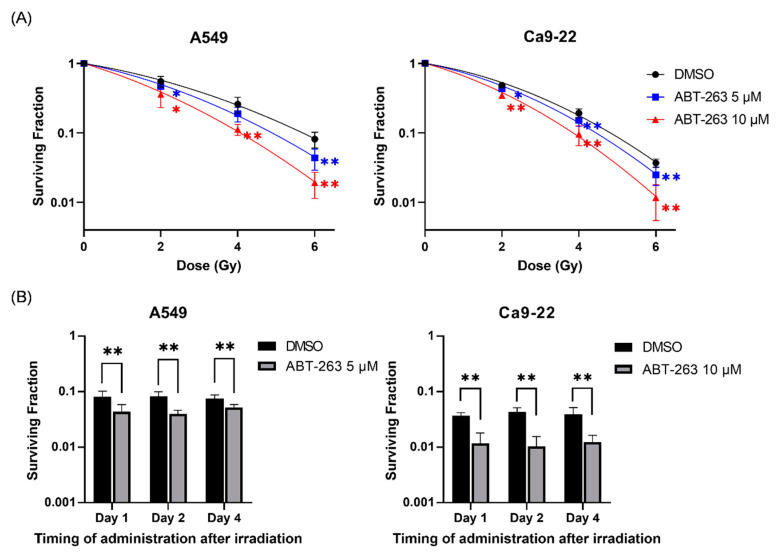
Effects of ABT-263 on the survival of irradiated cancer cells. (**A**) X-irradiated A549 and Ca9-22 cells were cultured for 24 h prior to the addition of ABT-263 to the culture. After further culture, the colony number was counted and the surviving fraction was calculated. (**B**) A549 and Ca9-22 cells were irradiated at 6 Gy. At 1, 2, and 4 days after irradiation, ABT-263 was added to the culture. After culture, the surviving fraction was calculated. Data are presented as the mean ± SD of independent experiments. * *p* < 0.05, ** *p* < 0.01.

Next, the effects of ABT-263 on the survival of irradiated A549 and Ca9-22 cancer cells were investigated. As shown in Figure 2A, the addition of ABT-263 to the culture 24 h after irradiation significantly decreased the surviving fraction of irradiated cancer cells compared with the vehicle control group (DMSO). The radiation dose where 10% of cells will survive (D_10_) of A549 was lower in the ABT-263 group (4.89 Gy for 5 μM and 4.06 Gy for 10 μM) compared with the DMSO group (5.69 Gy). Similarly, the D_10_ of Ca9-22 cells treated with ABT-263 (4.44 Gy for 5 μM and 3.85 Gy for 10 μM) was lower than that of the DMSO group (4.81 Gy). The ratio of D_10_ at 10 μM ABT-263 in A549 and Ca9-22 was 1.40 and 1.25, respectively, thus indicating that ABT-263 decreased the survival of irradiated cells. Furthermore, similar to the results of the administration of ABT-263 at 24 h (Day 1) after irradiation, the fraction of surviving cells irradiated at 6 Gy was also decreased by the addition of ABT-263 to the culture at two and four days after irradiation (Figure 2B).

**Figure 3:**
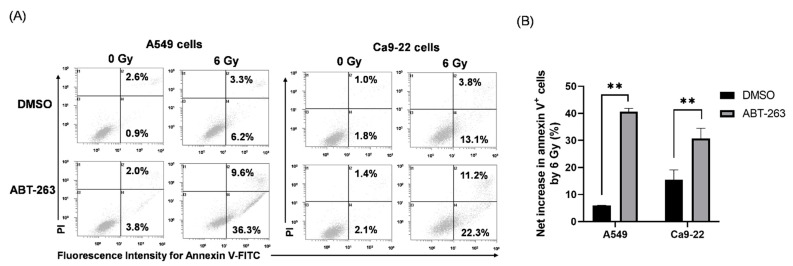
Effects of ABT-263 on the induction of apoptosis of irradiated cancer cells: (**A**,**B**) X-irradiated A549 and Ca9-22 cells were cultured for 24 h prior to the addition of ABT-263 to the culture (5 μM for A549 cells and 10 μM for Ca9-22 cells). After 24 h of culture, the cells including floating cells were harvested for apoptosis analysis. (**A**) Representative cytograms are shown. The inset numbers indicate the percentage of annexin V^+^/propidium iodine (PI)^−^ cells or annexin V^+^/PI^+^ cells. (**B**) The net increase in annexin V^+^ apoptotic cells (sum of annexin V^+^/PI^−^ cells and annexin V^+^/PI^+^ cells) by 6 Gy is shown. Data are presented as the mean ± SD of independent experiments. ** *p* < 0.01.

Senolytic drugs are known to eliminate senescent cells by inducing apoptosis [17]. Therefore, the effect of ABT-263 on the induction of apoptosis of irradiated cancer cells was also examined. As shown in Figure 3A, although the addition of ABT-263 hardly increased the proportion of annexin V^+^ apoptotic cells in non-irradiated cancer cells, the percentage of apoptotic ABT-263-treated irradiated cells was clearly higher than that of the DMSO-treated irradiated cells. Furthermore, there was a significantly greater net increase in apoptotic cells by 6 Gy-irradiation in the ABT-263 group compared to the DMSO group (Figure 3B). Taken together, these results suggest that administration of ABT-263 after irradiation decreased the survival of irradiated cancer cells.

### 2.3. Effect of Co-Culture with Senescence-like A549 Cells on the Survival of Non-Irradiated and Irradiated A549 Cells

As described above, ABT-263 administration after irradiation resulted in decreased survival of irradiated cancer cells accompanied by a decrease in the proportion of cells with high SA-β-gal activity. These results suggest that the existence of senescence-like cancer cells play an important role in the survival of irradiated cancer cells. Next, the involvement of senescence-like cancer cells in the survival of irradiated cancer cells was investigated.

**Figure 4:**
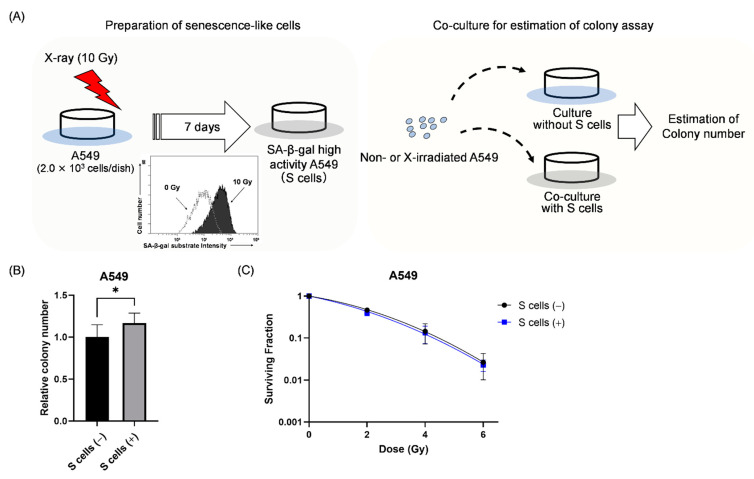
Effect of co-culture with senescence-like A549 cells on the survival of non- and irradiated A549 cells. (**A**) A schematic of the co-culture experiment is shown. A549 cells irradiated with 10 Gy were cultured for 7 days to induce senescence-like cells (S cells). Non-irradiated and X-ray irradiated A549 cells were seeded in dishes with or without S cells for the colony-forming assay. (**B**) The relative colony number of non-irradiated A549 cells in the presence and absence of S cells is shown. (**C**) The surviving fraction of A549 cells with and without S cells is shown. S cells (+) and S cells (−) indicate with and without S cells, respectively. Data are presented as the mean ± SD of independent experiments. * *p* < 0.05.

Accumulating evidence indicates that secretory substances resulting from the SASP contribute to cancer growth, progression, and recurrence [18,19]. Therefore, we hypothesized that senescence-like cancer cells support the growth of other irradiated cancer cells that do not show a senescence-like phenotype. To test this hypothesis, non- and X-irradiated A549 cells were co-cultured with senescence-like A549 cells (hereafter S cells) induced by 10 Gy (Figure 4A and Section 4.6). As shown in Figure 4B, when non-irradiated A549 cells were co-cultured with senescence-like cells, the colony number of non-irradiated A549 cells was significantly higher in the presence compared to the absence of S cells. In terms of the irradiated A549 cells, co-culture with S cells hardly affected the surviving fraction of A549 cells (Figure 4C). These results suggest that S cells affect the survival of non-irradiated A549 cells, but not irradiated A549 cells.

### 2.4. Relationship between the Cell Survival and the Cells with Senescence-Related Features in Irradiated A549 Cells

The results of the co-culture experiment suggest that it is unlikely that senescence-like cancer cells support the growth of other irradiated cancer cells. Chemotherapy-induced cellular senescence is reportedly reversible in some types of cancers, as the cells gain the ability to re-proliferate [20]. Therefore, we next hypothesized that the irradiated cancer cells showing senescence-related features potentially proliferate, which contributes to the survival of irradiated cancer cells. This hypothesis was tested by investigating the relationship between the cell survival and the cells with senescence-related features in irradiated A549 cells using ABT-263.

**Figure 5:**
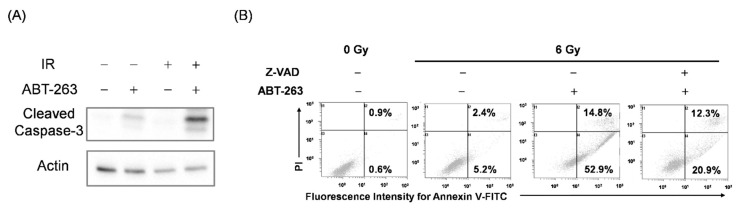
ABT-263 induces caspase-mediated apoptosis of irradiated A549 cells. (**A**) A549 cells irradiated at 6 Gy were cultured for 24 h prior to the addition of ABT-263 (5 μM) to the culture. After 24 h of culture, the cells were harvested for western blot analysis. β-actin was used as a loading control. Representative immunoblots are shown. (**B**) A549 cells irradiated at 6 Gy were cultured for 24 h prior to the addition of ABT-263 to the culture. Z-VAD was added to the culture 1 h before ABT-263 administration. After 48 h of culture, the cells including floating cells were harvested for apoptosis analysis. Representative cytograms are shown. The inset numbers of the cytograms indicate the percentage of annexin V^+^/PI^−^ cells or annexin V^+^/PI^+^ cells.

To test this hypothesis, it is necessary to regulate the elimination of irradiated cancer cells by ABT-263. Since ABT-263 promoted apoptosis of irradiated cancer cells (Figure 3), we focused on apoptosis inducing factor caspase. As shown in Figure 5A, ABT-263 dramatically induced the expression of cleaved caspase-3 in irradiated A549 cells compared with each treatment alone. Furthermore, treatment with the caspase inhibitor Z-VAD markedly reduced the proportion of ABT-263-induced annexin V^+^ apoptotic cells in the irradiated A549 cells (Figure 5B). These results indicate that ABT-263 induces caspase-mediated apoptosis of irradiated A549 cells.

**Figure 6:**
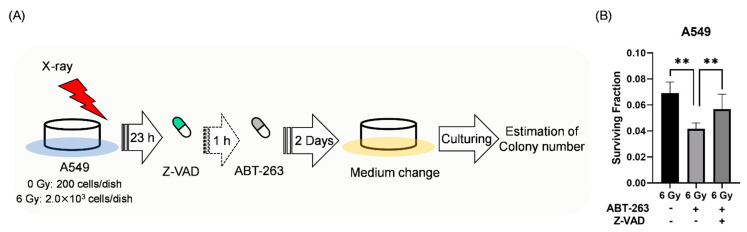
The caspase inhibitor Z-VAD attenuated the ABT-263-induced decrease in the surviving fraction of irradiated A549 cells. (**A**) The treatment procedure is shown. A549 cells irradiated at 6 Gy were cultured for 24 h prior to the addition of ABT-263 (5 μM) to the culture. After two days of culture, the medium containing Z-VAD and ABT-263 was replaced with fresh medium. The cultures were continued until colonies were formed. Then, the colony number was counted. (**B**) The surviving fraction of A549 cells is shown. Data are presented as the mean ± SD of independent experiments. ** *p* < 0.01.

Next, the effects of Z-VAD on the survival of irradiated A549 cells treated with ABT-263 were investigated. Since long-term inhibition of apoptosis could induce another form of programmed cell death [21], the irradiated A549 cells were treated with Z-VAD and ABT-263 for two days (Figure 6A). Similar to the results in Figure 2B, ABT-263 treatment for two days decreased the proportion of surviving irradiated A549 cells (Figure 6B). Of note, Z-VAD significantly recovered the ABT-263-induced decrease in the surviving fraction of irradiated A549 cells (Figure 6B). These results indicate that the cells eliminated by ABT-263 through caspase-mediated apoptosis contribute to the survival of irradiated A549 cells.

**Figure 7:**
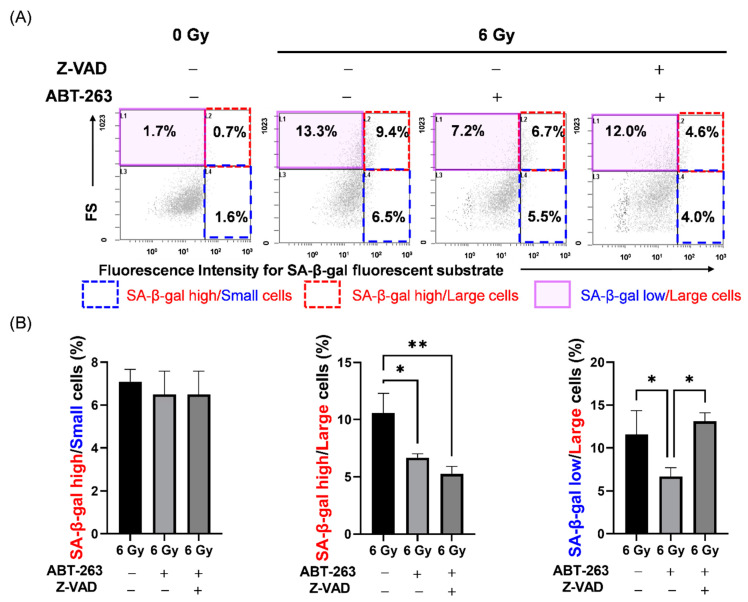
Effect of the caspase inhibitor Z-VAD on changes in cell populations of irradiated A549 cells by ABT-263. (**A**,**B**) A549 cells irradiated at 6 Gy were cultured for 24 h prior to the addition of ABT-263 (5 μM) to the culture. Z-VAD was added to the culture 1 h before ABT-263 administration. After 48 h of culture, SA-β-gal activity and FS, which indicates cell size, were analyzed by flow cytometry. (**A**) Representative cytograms of FS signals and fluorescence intensities of SA-β-gal are shown. The inset numbers of the cytograms indicate the percentage of SA-β-gal high/FS low (SA-β-gal high/small) cells (blue region), SA-β-gal high/FS high (SA-β-gal high/large) cells (red region), and SA-β-gal low/FS high (SA-β-gal low/large) cells (purple region). (**B**) The percentages of each population are shown. Data are presented as the mean ± SD of independent experiments. * *p* < 0.05, ** *p* < 0.01.

Next, the effects of Z-VAD and ABT-263 on the cell populations in irradiated A549 cells were investigated. Here, we focused on SA-β-gal activity and cell size as senescence-related features. Cell size was estimated by forward scatter (FS) signals with flow cytometry. As shown in Figure 7A, 6 Gy-irradiation increased the proportions of both SA-β-gal high-activity cells and large cells with high FS signals. Of note, although ABT-263 significantly reduced the populations of SA-β-gal high-activity/large and SA-β-gal low-activity/large cells in the irradiated A549 cells; only the SA-β-gal low-activity/large population was recovered by Z-VAD (Figure 7B). These results suggest that SA-β-gal low-activity/large cells may contribute to the survival of irradiated A549 cells.

**Figure 8:**
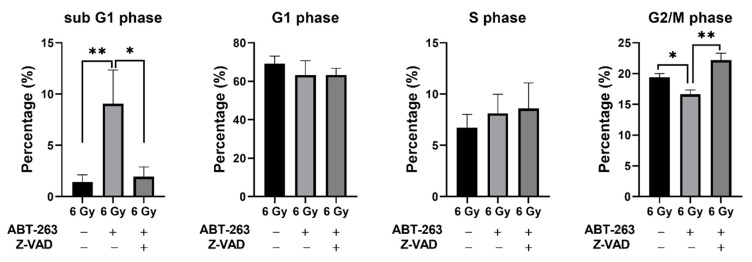
Effect of the caspase inhibitor Z-VAD on changes in the cell cycle distribution of irradiated A549 cells by ABT-263. A549 cells irradiated at 6 Gy were cultured for 24 h prior to the addition of ABT-263 (5 μM) to the culture. Z-VAD was added to the culture 1 h before ABT-263 administration. After 48 h of culture, the adherent cells were harvested for cell cycle analysis. The percentages of each population are shown. Data are presented as the mean ± SD of independent experiments. * *p* < 0.05, ** *p* < 0.01.

Cell size is an important indicator of cellular activity, as large cells contain relatively more cellular components such as proteins [22]. Furthermore, cell size is dependent on the cell cycle phase as cells in the G2/M phase are larger than in other phases [23]. Hence, SA-β-gal low-activity/large cells might indicate cell cycle progression. Therefore, the effect of Z-VAD on the changes in cell cycle distribution of irradiated A549 cells by ABT-263 was investigated. In accordance with the results of apoptosis analysis by annexin V staining, ABT-263 increased the sub G1 fraction, which is an apoptotic fraction, and Z-VAD had a significant suppressive effect. Of note, ABT-263 significantly reduced the G2/M population of irradiated A549 cells and Z-VAD recovered the population (Figure 8).

## 3. Discussion

Senolytic drugs are expected to be useful for the treatment of not only age-related diseases but also senescent cell-mediated adverse effects in cancer therapy [16]. However, the effects of senolytic drugs on the antitumor effects of cancer therapy remain unclear. In the present study, the effect of the senolytic drug ABT-263 on the survival of irradiated cancer cells was investigated. The results demonstrated that ABT-263 administration after irradiation decreased the survival of irradiated cancer cells, accompanied by a decrease in the fraction of cells with senescence-related features, suggesting that senescence-like cancer cells induced by irradiation contribute to the survival and proliferation of irradiated cancer cells. Therefore, the elimination of senescence-like cells in both normal and tumor tissues by ABT-263 could be beneficial for the treatment of adverse effects and enhancement of antitumor effects in radiation therapy.

Fractionated radiotherapy is in common use in clinical radiotherapy for cancers including head and neck squamous cell carcinoma [24]. In fractionated radiotherapy, irradiation to tumor (60–70 Gy) is usually performed by 2 Gy/day, five days/week. In the present study, we showed that the administration of ABT-263 after irradiation (even after four days) decreased the survival of irradiated cancer cells (Figure 2). Therefore, in the clinical radiation therapy, it is thought that administration of ABT-263 during or after a series of fractionated irradiation may be preferable.

As a potential mechanism of the contribution of senescence-like cancer cells induced by irradiation to the survival and proliferation of irradiated cancer cells, we hypothesized that the irradiated cells showing senescence-related features promote the proliferation of other irradiated cancer cells, as recent evidence suggests that senescent cells promote cancer growth [18,19]. However, the results of the co-culture experiment showed that the presence of S cells had no effect on the survival of irradiated A549 cells (Figure 4C). Therefore, it is unlikely that the irradiated cancer cells showing senescence-related features promote the proliferation of other irradiated cancer cells. However, intriguingly, co-culture with S cells increased the colony-forming ability of non-irradiated A549 cells (Figure 4B). It is known that senescent cells induce various effects such as cancer growth through SASP [19]. Interleukin-6 (IL-6), a proinflammatory cytokine, is a representative factor of SASP [25], and is known to promote the proliferation of cancer cells [26,27]. Therefore, there is a possibility that SASP-related factors including IL-6 from the cells showing senescence-related features, promote the proliferation of non-irradiated cancer cells. Although there was no difference in the survival of irradiated cells with or without S cells, Wu et al. reported that pre-treatment with IL-6 before irradiation induced radioresistance [28]. The experimental condition where co-culture with S cells was performed after irradiation in the present study may account for the discrepancy. Therefore, further studies are needed to determine whether SASP-related factors are actually involved in the increased proliferation of non-irradiated cells by senescence-like cells.

Senolytic drugs are known to eliminate senescence-like cell populations in normal cells by inducing apoptosis [13,29]. This may be due to crosstalk between senescence and the apoptotic pathway. For example, the p53–p21 pathway is involved in both senescence and apoptosis. After DNA damage, p21 induces cell cycle arrest, leading to cellular senescence [30], while it mainly protects cells from apoptosis through the inhibition of apoptosis-inducing proteins [31]. The results of the present study showed that ABT-263 induced caspase-mediated apoptosis of irradiated cancer cells. Since ABT-263 is an inhibitor of the anti-apoptotic protein Bcl-2 family, it is thought that ABT-263 induces caspase-mediated apoptosis of irradiated cancer cells by inhibiting Bcl-2. Intriguingly, although ABT-263 decreased the populations of cells with senescence-related features (i.e., SA-β-gal high-activity cells and large cells), the caspase inhibitor recovered only SA-β-gal low-activity/large cells, but not SA-β-gal high-activity/large cells. These results suggest that ABT-263 induces apoptosis of irradiated senescence-like cells in a caspase-dependent or -independent manner.

Importantly, the caspase inhibitor recovered not only the decreased survival of irradiated A549 cells by ABT-263 but also the decrease in the population of SA-β-gal low-activity/large cells by ABT-263. These results suggest that SA-β-gal low-activity/large cells eliminated by caspase-mediated apoptosis by ABT-263 may contribute to the survival of irradiated A549 cells. In addition, the caspase inhibitor recovered the decrease in cells in the G2/M phase, which signifies the preparation of cell division by inhibiting the ABT-263-induced sub G1 population may support this idea. In addition, distinguishable states in cellular senescence have been proposed and cellular morphological changes occur prior to induction of SA-β-gal high-activity [32]. Therefore, it is likely that SA-β-gal low-activity/large cells maintain the ability to proliferate due to a relatively early status of cellular senescence compared with SA-β-gal high-activity/large cells. Hence, future studies are warranted to investigate whether SA-β-gal low-activity/large cells actually proliferate.

In this study, SA-β-gal activity and cellular size were evaluated as senescence-related features. SA-β-gal activity is most frequently used for the detection of senescent cells, but is not an absolute marker. Therefore, further analysis of other senescence-related features such as factors related to the regulation of cell cycle arrest and SASP is needed to clarify cell populations with proliferative ability in irradiated cancer cells.

## 4. Materials and Methods

### 4.1. Reagents

Ca^2+^, Mg^2+^-free Dulbecco’s phosphate-buffered saline PBS(−) was purchased from Wako Pure Chemical Industries, Ltd. (Osaka, Japan). PI and dimethyl sulfoxide (DMSO) were purchased from Sigma-Aldrich (St. Louis, MO, USA). ABT-263 and Z-Val-Ala-Asp(OMe)-CH2F (3188-v, Z-VAD) were purchased from Selleck Chemicals (Houston, TX, USA) and Peptide Institute Inc. (Osaka, Japan), respectively. Annexin V binding buffer and fluorescein isothiocyanate (FITC)-labeled annexin V (FITC-annexin V) were purchased from BioLegend (San Diego, CA, USA). Senescence β-Galactosidase Activity Assay Kit (#35302), cleaved caspase-3 antibody (#9661), β-actin antibody (#4967), and horseradish peroxidase (HRP)-labeled anti-rabbit IgG antibody (#7074) were purchased from Cell Signaling Technology Japan, K.K. (Tokyo, Japan).

### 4.2. Cell Culture and Treatment

Human lung adenocarcinoma A549 cells and human head and neck squamous cell carcinoma Ca9-22 cells were obtained from the RIKEN Bio-Resource Research Center (Tsukuba, Japan). A549 cells were maintained in low-glucose Dulbecco’s modified Eagle’s medium (DMEM; Sigma-Aldrich) supplemented with 10% heat-inactivated fetal bovine serum (FBS; Sigma-Aldrich) and 1% penicillin/streptomycin (P/S; Wako Pure Chemical Industries). Ca9-22 cells were maintained in high-glucose DMEM (Wako Pure Chemical Industries) supplemented with 1% P/S and 10% FBS. Both cell lines were cultured at 37 °C under a humidified atmosphere of 5% CO_2_/95% air.

Cells (1.0 × 10^5^) were seeded in 35-mm culture dishes (Sumitomo Bakelite Co. Ltd., Tokyo, Japan) and incubated for 6 h to allow adherence to the dishes. After incubation, the cells were exposed to X-ray. After culturing for 24 h, ABT-263 was added to the culture medium. In some experiments, Z-VAD (20 μM) was added to the culture medium 1 h before ABT-263 administration. DMSO-treated cells were prepared as a vehicle control for ABT-263 and Z-VAD. The cells were cultured for the indicated time periods at 37 °C and then harvested using 0.1% trypsin-ethylendiaminetetraacetatic acid (Wako Pure Chemical Industries) for subsequent analysis.

### 4.3. In Vitro X-ray Irradiation

X-ray irradiation (150 kVp, 20 mA, 0.5 mm Al, and 0.3 mm Cu filters) was performed using an X-ray generator (MBR-1520R-3; Hitachi Medical Corporation, Tokyo, Japan) at a distance of 45 cm from the focus and dose rate of 1.00–1.03 Gy/min.

### 4.4. Analysis of SA-β-Gal Activity

SA-β-gal activity was analyzed using the Senescence β-Galactosidase Activity Assay Kit according to the manufacturer’s instructions. In brief, after treatment, the culture medium was replaced with culture medium containing bafilomycin A1 (100 nM). After incubation for 1 h at 37 °C, SA-β-Gal substrate solution (33 µM) was added to the cell culture, which was further incubated for 2 h at 37 °C. After washing three times with PBS(−), the cells were harvested, washed with PBS(−), and suspended in cold PBS(−) containing 2% FBS. The fluorescence intensity of the SA-β-Gal substrate was analyzed using a flow cytometer (Cytomics FC500; Beckman Coulter, Inc., Brea, CA, USA).

### 4.5. Clonogenic Survival Assay

Cells were seeded in 60-mm culture dishes (Sumitomo Bakelite Co. Ltd.) and incubated for 6 h to allow them to adhere to the dishes. After incubation, the cells were exposed to X-ray irradiation. After culturing for 24, 48, or 96 h, ABT-263 was added to the culture medium. The cells were cultured for 7–13 days, fixed with methanol, and stained with Giemsa solution (Wako Pure Chemical Industries). Experiments were performed in triplicate. Colonies containing more than 50 cells were counted. The surviving fraction was calculated using GraphPad Prism 9 (GraphPad Software, Inc., San Diego, CA, USA) as previously reported [33].

### 4.6. Co-Culture Experiment

Prior to co-culture, A549 cells (2.0 × 10^3^) seeded in 60-mm culture dishes were irradiated with 10 Gy and cultured for one week to prepare senescence-like cells (S cells). After culturing, the dishes were washed with PBS(−), and the appropriate number of non- or X-irradiated cells were seeded in 60-mm culture dishes with S cells for co-culture. The cells were also seeded in new 60-mm culture dishes without S cells. After culturing for 7–9 days, the colonies were fixed, stained, and counted as described above. The dishes containing only S cells were also prepared to count the colony number derived from S cells. The number of S cell colony dishes was subtracted from that of the co-culture dishes. Experiments were performed in triplicate.

### 4.7. Apoptosis Analysis

Apoptosis analysis was performed using FITC-annexin V and PI, as previously reported [34]. Annexin V^+^ cells (sum of annexin V^+^/PI^−^ and annexin V^+^/PI^+^ cells) were defined as apoptotic cells.

### 4.8. Sodium Dodecyl Sulfate—Polyacrylamide Gel Electrophoresis (SDS-PAGE) and Western Blot Analysis

SDS-PAGE and western blot analysis were performed as previously reported [35]. The samples were probed with primary antibodies against cleaved caspase-3 (1:3000) and β-actin (1:4000), followed by HRP-conjugated anti-rabbit secondary antibodies against IgG (1:10,000). The antigens were visualized using Clarity MAX^TM^ Western ECL Substrate (Bio-Rad Laboratories, Inc., Hercules, CA, USA).

### 4.9. Cell Cycle Analysis

Cell cycle analysis was performed as previously reported [36]. In brief, the treated cells were harvested, fixed with 70% ethanol, treated with RNase (Sigma-Aldrich), and stained with PI. Cell cycle distribution was analyzed using a flow cytometer.

### 4.10. Statistical Analysis

Data are presented as the mean ± SD of at least three independent experiments. Statistical analyses were performed using Excel 2016 software (Microsoft Corporation, Redmond, WA, USA) along with the add-in software Statcel v4 (OMS publishing Inc., Tokyo, Japan) or GraphPad Prism v9 (GraphPad Software, Inc.). A probability (*p*) value of <0.05 was considered statistically significant.

## 5. Conclusions

In conclusion, the present study demonstrated that ABT-263 administration after irradiation decreases the survival of irradiated cancer cells. Furthermore, populations maintaining proliferative ability exist among the irradiated cancer cells showing senescence-related features, which contributes to the survival of irradiated cancer cells.

## Data Availability

The data presented in this study are available in the article.

## Supplementary Materials

The following are available online at https://www.mdpi.com/article/10.3390/ijms222413233/s1.
