# Supplementary material for: Effects and Related Mechanisms of the Senolytic Agent ABT-263 on the Survival of Irradiated A549 and Ca9-22 Cancer Cells
Source: Int J Mol Sci. 2021 Dec 8;22(24):13233. doi: 10.3390/ijms222413233 (PMC8704639; doi:10.3390/ijms222413233)
Supplement: Supplementary file 1 [file ijms-22-13233-s001.zip › ijms-1497180-supplementary.pdf]

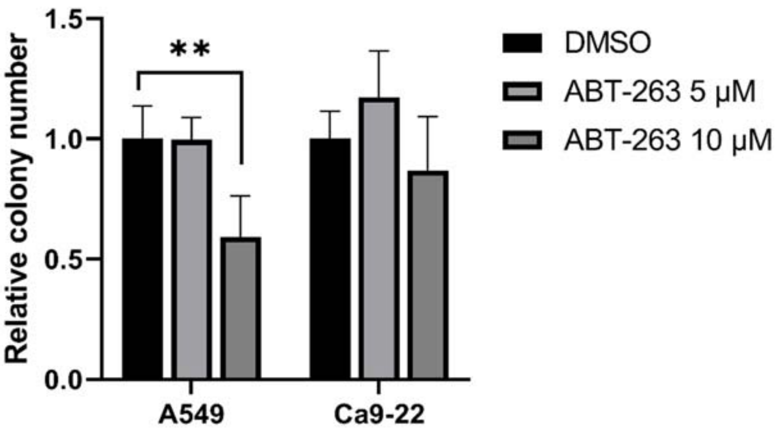

Figure S1. Effect of ABT-263 on colony-forming ability of non-irradiated cancer cells: The colony-forming ability of A549 and Ca9-22 cells in the presence of 5 or 10 μM ABT-263 was examined. The results of relative colony number compared with dimethyl sulfoxide (DMSO) is shown. Data are presented as the mean ± standard deviation of independent experiments. **$p < 0.01$.
